# Measurement of ultra‐fast signal progression related to face processing by 7T fMRI

**DOI:** 10.1002/hbm.24907

**Published:** 2020-01-10

**Authors:** Uk‐Su Choi, Yul‐Wan Sung, Seiji Ogawa

**Affiliations:** ^1^ Center for Information and Neural Networks National Institute of Information and Communications Technology Osaka Japan; ^2^ Graduate School of Frontier Biosciences Osaka University Osaka Japan; ^3^ Kansei Fukushi Research Institute Tohoku Fukushi University Sendai Japan; ^4^ Neuroscience Research Institute Gachon University Incheon Korea

**Keywords:** fMRI, interhemispheric transfer time, inter‐stimulus interval

## Abstract

Given that the brain is a dynamic system, the temporal characteristics of brain function are important. Previous functional magnetic resonance imaging (fMRI) studies have attempted to overcome the limitations of temporal resolution to investigate dynamic states of brain activity. However, finding an fMRI method with sufficient temporal resolution to keep up with the progress of neuronal signals in the brain is challenging. This study aimed to detect between‐hemisphere signal progression, occurring on a timescale of tens of milliseconds, in the ventral brain regions involved in face processing. To this end, we devised an inter‐stimulus interval (ISI) stimulation scheme and used a 7T MRI system to obtain fMRI signals with a high signal‐to‐noise ratio. We conducted two experiments: one to measure signal suppression depending on the ISI and another to measure the relationship between the amount of suppression and the ISI. These two experiments enabled us to measure the signal transfer time from a brain region in the ventral visual stream to its counterpart in the opposite hemisphere through the corpus callosum. These findings demonstrate the feasibility of using fMRI to measure ultra‐fast signals (tens of milliseconds) and could facilitate the elucidation of further aspects of dynamic brain function.

## 1 INTRODUCTION

Most functional magnetic resonance imaging (fMRI) studies have explored brain function from a static perspective, identifying brain areas with functional selectivity and specificity. Brain mapping research has yielded considerable information on static functional relationships between brain regions, including the hierarchical structures of the brain in the ventral visual pathway and the pathway from the occipital to parietal lobes, including the hemispheric structure. Such research has provided a strong basis to understand brain function from the perspective of functional localization; many brain areas have been shown to perform specific information processing tasks, including processing faces, objects, and scenes, for example (Epstein & Kanwisher, 1998; Grill‐Spector, 2003; Yovel & Kanwisher, 2004).

However, the brain is not a static system. Brain dynamics arise from the flow of signals, such as the transforming of timed inputs to timed outputs, either within or between brain areas (Ishizu, Ayabe, & Kojima, 2009; Yuste & Fairhall, 2015; Zhang, Zhu, & Chen, 2008) where neural activities are encoded with precise time control (Sehatpour et al., 2008; Stigliani, Jeska, & Grill‐Spector, 2017). According to recent resting‐state fMRI studies, the dynamic changes in the brain are vital to both information processing and dealing with constantly changing environments (Deco, Kringelbach, Jirsa, & Ritter, 2017; Mitra et al., 2018; Mitra, Snyder, Blazey, & Raichle, 2015; Mitra, Snyder, Hacker, & Raichle, 2014). Studies have shown that specific brain states can be encoded by temporal information. For example, one study has found that signals from the frontal areas of the brain are sent to the posterior areas under anesthesia, and the signals are sent in the reverse direction when the subject is conscious (Mitra et al., 2018). Brain states may change dynamically during task processing. Therefore, understanding both the dynamics of the brain and the static characteristics of the functional selectivity and specificity of brain areas are essential in understanding the mechanisms of brain function (Hauk, 2016). These dynamic changes can be investigated using task‐fMRI.

The measurement of the transfer signals between brain areas is key to understanding brain dynamics. Electroencephalography (EEG) and magnetoencephalography (MEG) have been used to measure the interhemispheric transfer time (IHTT) between the right and left hemispheres (Barca et al., 2011; Bayard, Gosselin, Robert, & Lassonde, 2004; Nalcaci, Basar‐Eroglu, & Stadler, 1999). These technologies measure transfer times as latency differences between the right and left hemispheres and can achieve a resolution of tens of milliseconds; however, they have poor spatial resolution, and thus cannot provide reliable information on the spatial specificity of brain functions. Therefore, using these methods, it is difficult to measure specific transfer times either between areas in different hemispheres or between homotopic brain areas. Taking advantage of the high spatial resolution of fMRI, several recent studies have examined brain dynamics by evaluating oscillatory fMRI responses and deconvolution of the blood oxygenation level‐dependent (BOLD) response in the visual pathway (Lewis, Setsompop, Rosen, & Polimeni, 2016, 2018). However, the current resolution of the latter approaches is only several seconds.

Therefore, an alternative approach is needed to measure fast signal transfer between regions of interest (ROIs) in the brain. In case of fMRI, a paired‐stimulus paradigm can be used to measure neuronal activity at the scale of several tens of milliseconds. The paired‐stimulus paradigm has been used for measuring inter‐stimulus interval (ISI)‐dependent suppression of BOLD signals in both the rat somatosensory cortex and the human visual cortex, revealing fast neuronal processing at ~40 and 200 ms, respectively (Ogawa et al., 2000). In addition, the paired‐stimulus paradigm has been applied for the detection of fast neuronal interactions at 300 ms between auditory and visual modalities through ISI‐dependent suppression of BOLD signals (Zhang & Chen, 2006; Zhang, Zhu, & Chen, 2005). In another study, the paired‐stimulus paradigm with various ISIs was used to elucidate inhibitory interactions at less than 100 ms between ocular dominance columns (Zhang, Zhu, Yacoub, Ugurbil, & Chen, 2010).

Recent fMRI studies using ultra‐high field MRI have produced higher signal‐to‐noise ratios (SNRs) than previous work. Thus, this approach has higher functional sensitivity (Pohmann, Speck, & Scheffler, 2016; Triantafyllou et al., 2005), which could help identify small differences in BOLD signals under similar task conditions in fMRI studies.

**Figure 1:**
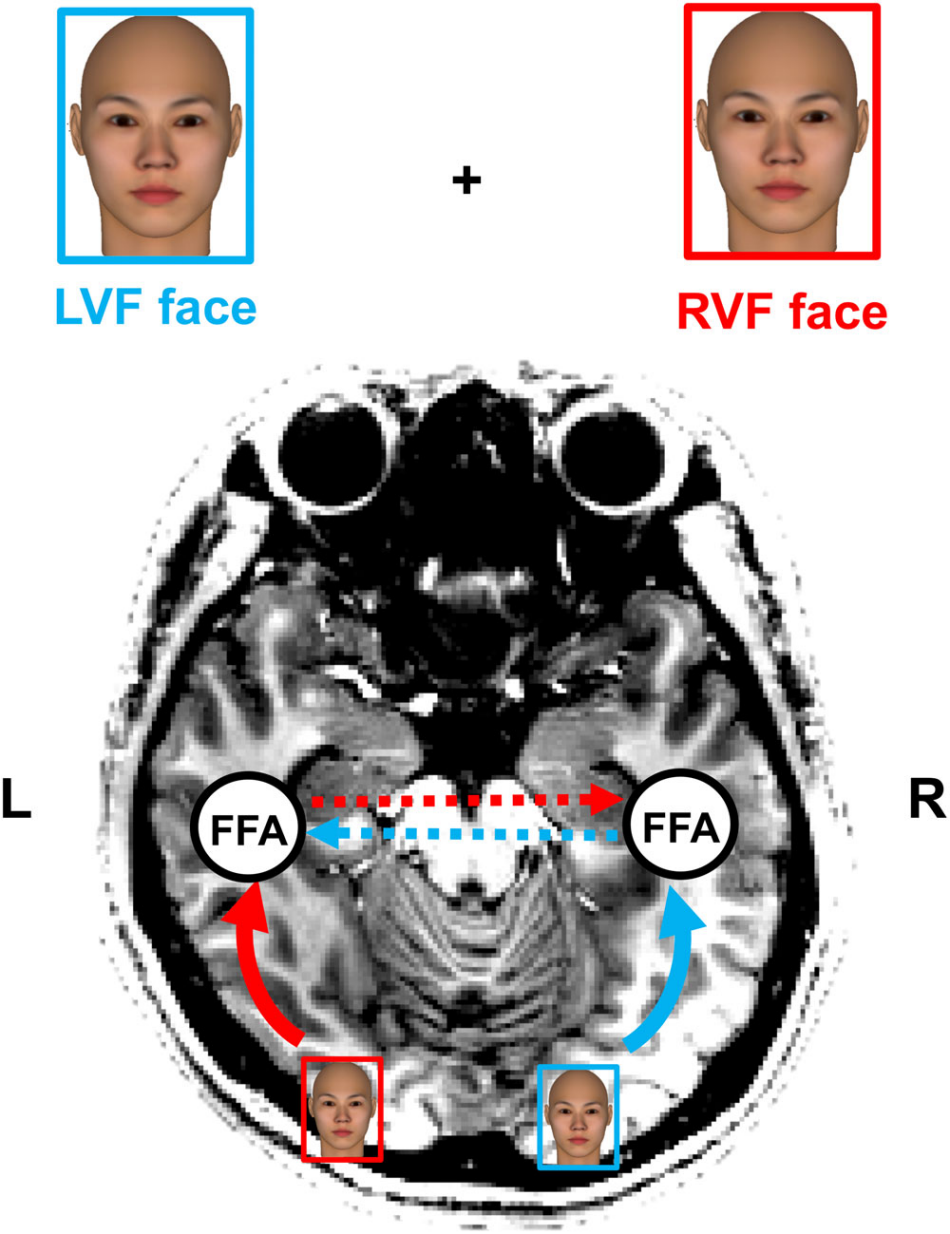
Flow of signal input to FFA from the visual fields. A face shown in the left visual field is projected into the right V1, and the signal is passed up to the right FFA (sky‐blue solid line) and also transferred to the left FFA (sky‐blue dotted line). In the same way, the face shown in the right visual field is projected into the left V1 and then passed up to the left FFA (red solid line) and the right FFA (red dotted line). LVF indicates the left visual hemifield and RVF indicates the right visual hemifield. FFA, fusiform face area

**Figure 2:**
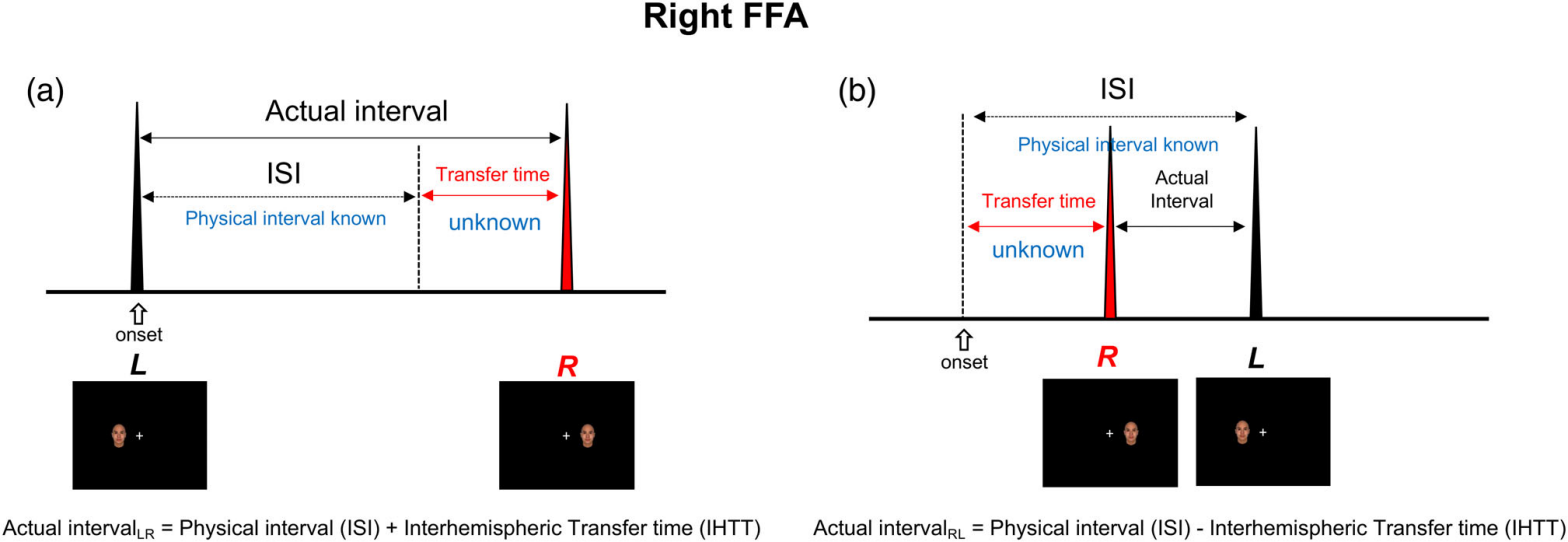
A model of the actual interval between two stimuli (L and R), as seen at the right FFA. Parts (a) and (b) show the actual intervals of conditions LR and RL, respectively. The actual intervals at the right FFA vary depending on the order of stimulation in the stimulus pair because of the transfer time (IHTT) between hemispheres. The actual interval for LR is longer (a) than that for condition RL (b). As the physical interval (ISI) is the same, the subtraction of the actual interval for condition RL from the actual interval for condition LR becomes 2*IHTT in this model

This study aimed to build upon previous studies in measuring the transfer times between specific brain areas in the right and left hemispheres by applying the paired‐stimulus paradigm to signal transfer between face‐processing areas and to repetition suppression in the face‐processing areas. As is well known, a unilaterally applied stimulus evokes only its contralateral primary visual area (V1), and the activated signal is then transmitted laterally to higher‐tier areas, such as the occipital face area (OFA) and the fusiform face area (FFA), while some of the signal crosses the corpus callosum (CC) to the other hemisphere (Hemond, Kanwisher, & Op de Beeck, 2007). We also observed this kind of signal transfer between hemispheres and found greater signal transfer in the FFA than in the OFA (Choi et al., 2013). There was little difference between the activation amplitude in the FFA in response to a stimulus presented at its ipsilateral visual field and the response to the same stimulus presented at the contralateral visual field. These characteristics were also observed in repetition suppression in the FFA. Repetition suppression has been observed both by using repeated face stimuli presented in the same visual field and by the successive stimulation of one visual field, followed by the stimulation of the other visual field after a short ISI (Sung et al., 2010). Irrespective of the visual field, the FFA receives the same face information (Figure 1), and the same suppression occurs at the FFA irrespective of differences in the visual field. We devised a stimulus scheme (Figure 2 and the Section 2) and measured the transfer time between hemispheres (Figure 2) as follows: (a) the measurement of repetition suppression of paired stimuli presented bilaterally at the right and left visual fields, with a specified ISI; (b) the measurement of repetition suppression of paired stimuli presented unilaterally at the same visual field; (c) the derivation of a relationship between the amount of suppression and the ISI of the paired stimuli at (b) (this metric quantifies the amount of suppression in relation to ISI); and (d) the estimation of transfer times between hemispheres from the suppression at (a) through the relationship between the amount of suppression and the ISI obtained at (c).

## 2 METHODS

### 2.1 Participants

Two different subject groups participated in two different experiments. The first group comprised 22 healthy subjects (11 males and 11 females) (age [mean ± *SD*] 22.74 ± 1.58 years), who participated in the first experiment. The second subject group comprised 12 healthy subjects (8 males and 4 females) (age [mean ± *SD*] 22.42 ± 1.24 years), who participated in the second experiment. All subjects were provided with information about the study and provided written informed consent, in accordance with the Declaration of Helsinki. This study was approved by the Institutional Review Board of Gachon University.

### 2.2 Study design

**Figure 3:**
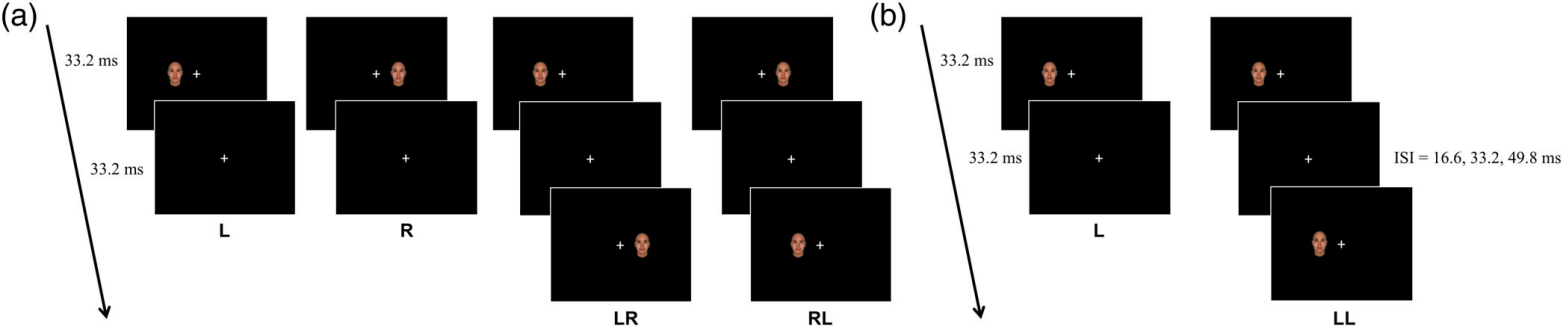
Stimulus conditions in the first experiment (a) and the second experiment (b). (a) Four different conditions: L (single face stimulation in the left visual field); R (single face stimulation in the right visual field); LR (paired stimuli, first face stimulation in the left visual field and second face stimulation in the right visual field, with an ISI of 33.2 ms); and RL (paired stimuli, first face stimulus in the right visual field and second face stimulus in the left visual field, with an ISI of 33.2 ms). (b) Four different conditions: L (single face stimulus in the left visual field) and LLs (paired face stimuli presented at the same location of the left visual field with ISIs of 16.6, 33.2, and 49.8 ms). ISI, inter‐stimulus interval

Two main experiments and an additional localization experiment were performed. The localization experiment was a block design consisting of two stimuli conditions. Face images were generated artificially using FaceGen (Singular Inversions, Toronto, Canada) and presented with 60‐Hz refresh rates through a projector (Panasonic, PT‐D5500, Osaka, Japan). E‐prime (Psychology Software Tools, Sharpsburg, PA) was used for stimulus presentation. The presentation time was controlled based on frame unit using options in the E‐prime (prelease and onset synchronization of a vertical blank), and the frame time was rounded off up to one decimal place. Each stimulation condition consisted of three blocks of 12 s duration, and each block consisted of eight trials each lasting 1.5 s. There were rest blocks of 18 s duration between stimulus condition blocks, before the first stimulus condition block, and after the last stimulus condition block. The total time of the localization scan was 180 s. The first experiment consisted of four conditions with different visual fields: L (single face stimulus in the left visual field); R (single face stimulus in the right visual field); LR (paired stimuli, first face stimulus in the left visual field and second face stimulus in the right visual field); and RL (paired stimuli, first face stimulus in the right visual field and second face stimulus in the left visual field) with a 33.2‐ms ISI (two frames of 60‐Hz refresh rate, 33.2 ms). The stimulus duration of face images was 33.2 ms, and the inter‐trial period was 1,500 ms, due to a rest period being added following each trial for paired stimuli. There were eight trials, each of 12 s duration, in each stimulation block. Stimulation blocks were repeated three times for each stimulus condition. The faces used in the trials were different from each other in each block of the experiments, that is, the single stimulus block used eight different faces and the paired‐stimulus block had eight different pairs of faces. At the start of the scan, a dummy block, which was excluded from the analysis, was inserted to avoid any effects due to the abrupt initiation of stimulation. A rest block was inserted between stimulus condition blocks and after the last stimulus condition block. The experimental run lasted 390 s, which included a dummy block of 12 s and the rest blocks of 18 s (Figure 3a).

The second experiment consisted of four conditions: L (single face stimulus in the left visual field); LL16.6 (paired face stimuli with 16.6‐ms ISI in the left visual field); LL33.2 (paired face stimuli with 33.2‐ms ISI in the left visual field); and LL49.8 (paired face stimuli with 49.8‐ms ISI in the left visual field) (Figure 3b). The stimulus duration of the face images was 33.2 ms. The trial interval was 1,500 ms, due to a rest period being added after each trial. Eight trials in each stimulation block brought the duration of each block to 12 s. Each block was repeated three times during the experimental run for each stimulus condition. The faces used in the trials were different from each other in each block of the experiments, that is, the single stimulus block used eight different faces and the paired‐stimulus block had eight different pairs of faces. At the start of the scan, a dummy block, which was excluded from the analysis, was inserted to avoid any effects caused by the abrupt initiation of stimulation. A rest block of 18 s was inserted between stimulus blocks and after the last stimulus block. The experimental run lasted 390 s, which included a dummy block of 12 s and rest blocks of 18 s.

The first group, consisting of 22 subjects, participated in the first experiment and the localization experiment, and the second group, consisting of 12 subjects, participated in the second experiment and the localization experiment.

### 2.3 MRI measurements

The experiments were performed using a 7T MRI scanner with an eight‐channel matrix head coil (Magnetom, Siemens, Erlangen, Germany). Functional data were acquired by a gradient echo single‐shot echo planar image sequence (TR = 3,000 ms, TE = 29 ms, field of view = 192 × 192 mm, matrix = 192 × 192, in‐plane resolution = 1 × 1 mm, slice thickness = 1 mm, and number of slices = 35, GRAPPA acceleration factor = 2, partial Fourier = 7/8, flip angle = 88°). After functional images were acquired, anatomical images were acquired by a T1‐weighted image sequence (MPRAGE; magnetization prepared rapid gradient echo) (TR = 2,500 ms, TE = 3.01 ms, in‐plane resolution = 1 × 1 mm, slice thickness = 1 mm). Subjects were asked to identify the color of the cross‐hairs, which varied randomly, appearing at the center of the screen.

### 2.4 Data analysis

Functional and structural data were processed using BrainVoyager QX (Brain Innovation B.V., Postbus, city, the Netherlands). Functional data were preprocessed with motion correction, scan time correction, and high‐pass filtering with a cutoff frequency of 0.01 Hz, and without spatial smoothing. After preprocessing, the data were registered to native individual anatomical images, and a general linear model was applied to acquire brain activation maps. The FFA and OFA were defined by the contrast between the face condition and the building condition (*p* < .05, FDR corrected) from the localization experiment. Beta values from the first and second experiments were then extracted from these ROIs by convolving the predictor of stimulus conditions with the hemodynamic response function that is incorporated in BrainVoyager QX. Beta values from the FFA, OFA, and V1 were extracted from each individual subject, and a one‐way analysis of variance with four conditions and subjects was conducted using SPSS 20 (SPSS Inc., Chicago, IL). The statistical significance of the differences between conditions was calculated by conducting a multiple‐comparison analysis. The repetition suppression ratio in the first experiment was calculated using the following Equation ((1)) for RL stimulation and Equation (2) for LR stimulation:

(1) $$\text{Suppression ratio}=\frac{\left({\text{beta}}_{L}+{\text{beta}}_{R}\right)-{\text{beta}}_{\mathrm{RL}}}{\left({\text{beta}}_{L}+{\text{beta}}_{R}\right)}\times 100$$

(2) $$\text{Suppression ratio}=\frac{\left({\text{beta}}_{L}+{\text{beta}}_{R}\right)-{\text{beta}}_{\mathrm{LR}}}{\left({\text{beta}}_{L}+{\text{beta}}_{R}\right)}\times 100$$

The repetition suppression ratio in the second experiment was also calculated using the following Equation ((3)):

(3) $$\text{Suppression ratio}=\frac{2\times \left({\text{beta}}_{L}\right)-{\text{beta}}_{\mathrm{LL}}}{2\times \left({\text{beta}}_{L}\right)}\times 100$$

The beta values of the paired stimuli in the first experiment were normalized to those in the second experiment using the following equation to directly compare the responses to paired stimuli of the two different experiments.

The repetition suppression of bilaterally presented stimuli corresponding to the physical interval (ISI) effect (that is, using a transfer time of zero) was also estimated. The actual interval for LR (actual interval_LR) was the physical interval plus the transfer time, and the actual interval for RL (actual interval_RL) was the physical interval minus the transfer time (Figure 2). From these values, the physical interval without the transfer time could be derived as:

$$\left(\text{actual interval}\_\mathrm{LR}+\text{actual interval}\_\mathrm{RL}\right)/2$$

The BOLD responses for LR and RL are for the actual interval_LR and actual interval_RL. Therefore, the BOLD response for the physical interval LR/RL is:

$$\left(\text{BOLD response}\_\mathrm{LR}+\text{BOLD response}\_\mathrm{RL}\right)/2$$

The repetition suppression ratio to bilaterally presented stimuli depending on only the physical interval (ISI) was calculated from Equations (4) and (5).

(4) $${\text{beta}}_{\text{physical interval}\;\left(\mathrm{ISI}\right)}=\frac{{\text{beta}}_{\mathrm{LR}}+{\text{beta}}_{\mathrm{RL}}}{2}$$

(5) $${\text{Suppression ratio}}_{\text{physical interval}\;\left(\mathrm{ISI}\right)}=\frac{\left({\text{beta}}_{L}+{\text{beta}}_{R}\right)-{\text{beta}}_{\text{Physical interval}\;\left(\mathrm{ISI}\right)}}{\left({\text{beta}}_{L}+{\text{beta}}_{R}\right)}\times 100$$

A one‐sample *t* test was performed to estimate mean values (suppression ratio, differences of suppression ratio, and the IHTT) across subjects. When running these one‐sample *t* tests in SPSS 20, the 95% confidence intervals of *t*‐statistics were also computed to estimate the inter‐subject variations.

## 3 RESULTS

The transfer time was measured according to subsections 3.2‐3.5 following the localization of face areas.

### 3.1 Localization of face areas

**Figure 4:**
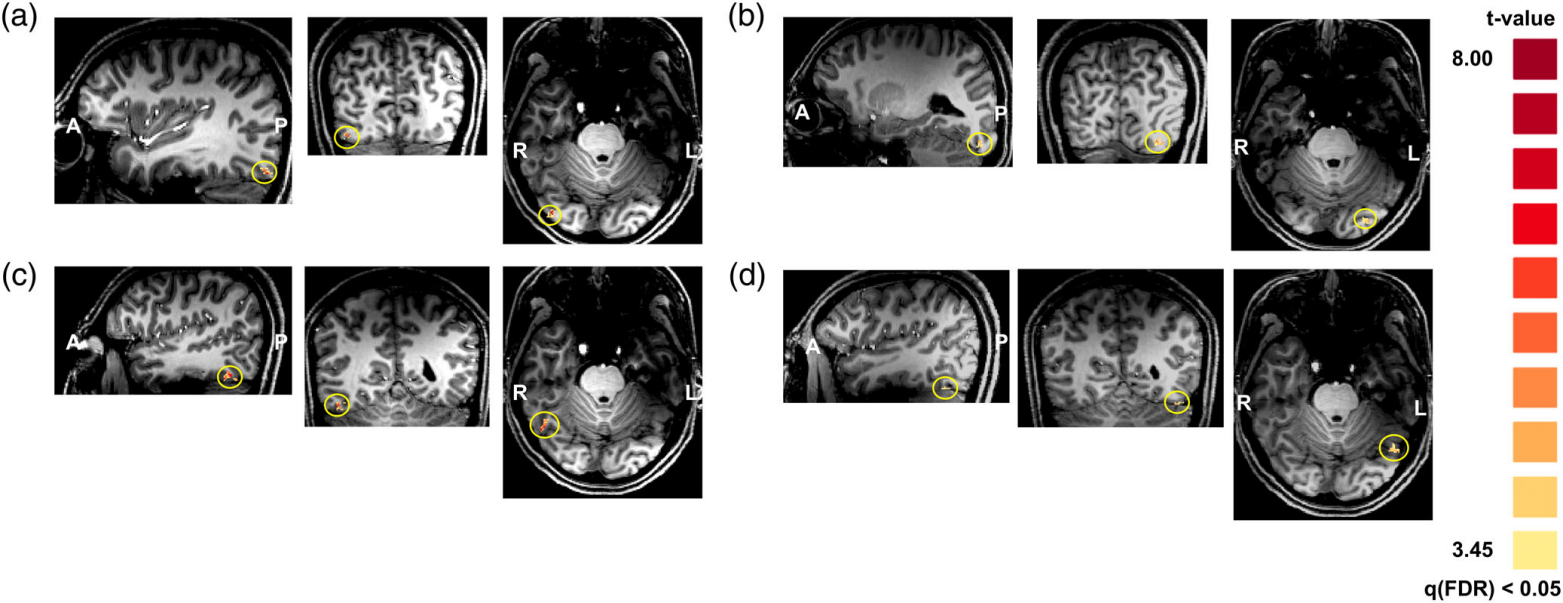
Target ROIs of the right OFA (a), the left OFA (b), the right FFA (c), and the left FFA (d), which were identified by the localization experiment: an example from one subject. In the first experiment, the right FFAs from 22 subjects, the left FFAs from 21 subjects, the right OFAs from 13 subjects, and the left OFAs from 13 subjects were identified (*p* < .05, FDR corrected). In the second experiment, the right FFAs from 12 subjects, the left FFAs from 11 subjects, the right OFAs from 12 subjects, and the left OFAs from nine subjects were identified (*p* < .05, FDR corrected). FFA, fusiform face area; OFA, occipital face area

Face‐processing areas, including the FFAs and OFAs in the right and the left hemispheres, were localized for the following ROI‐based analyses (Figure 4). In the first experiment, right FFAs from 22 subjects, left FFAs from 21 subjects, right OFAs from 13 subjects, and left OFAs from 13 subjects were identified (*p* < .05, false discovery rate [FDR] corrected). In the second experiment, right FFAs from 12 subjects, left FFAs from 11 subjects, right OFAs from 12 subjects, and left OFAs from 9 subjects were identified (*p* < .05, FDR corrected).

### 3.2 Measurement of repetition suppression of paired stimuli bilaterally presented at the right and left visual fields, with a specified ISI

**Figure 5:**
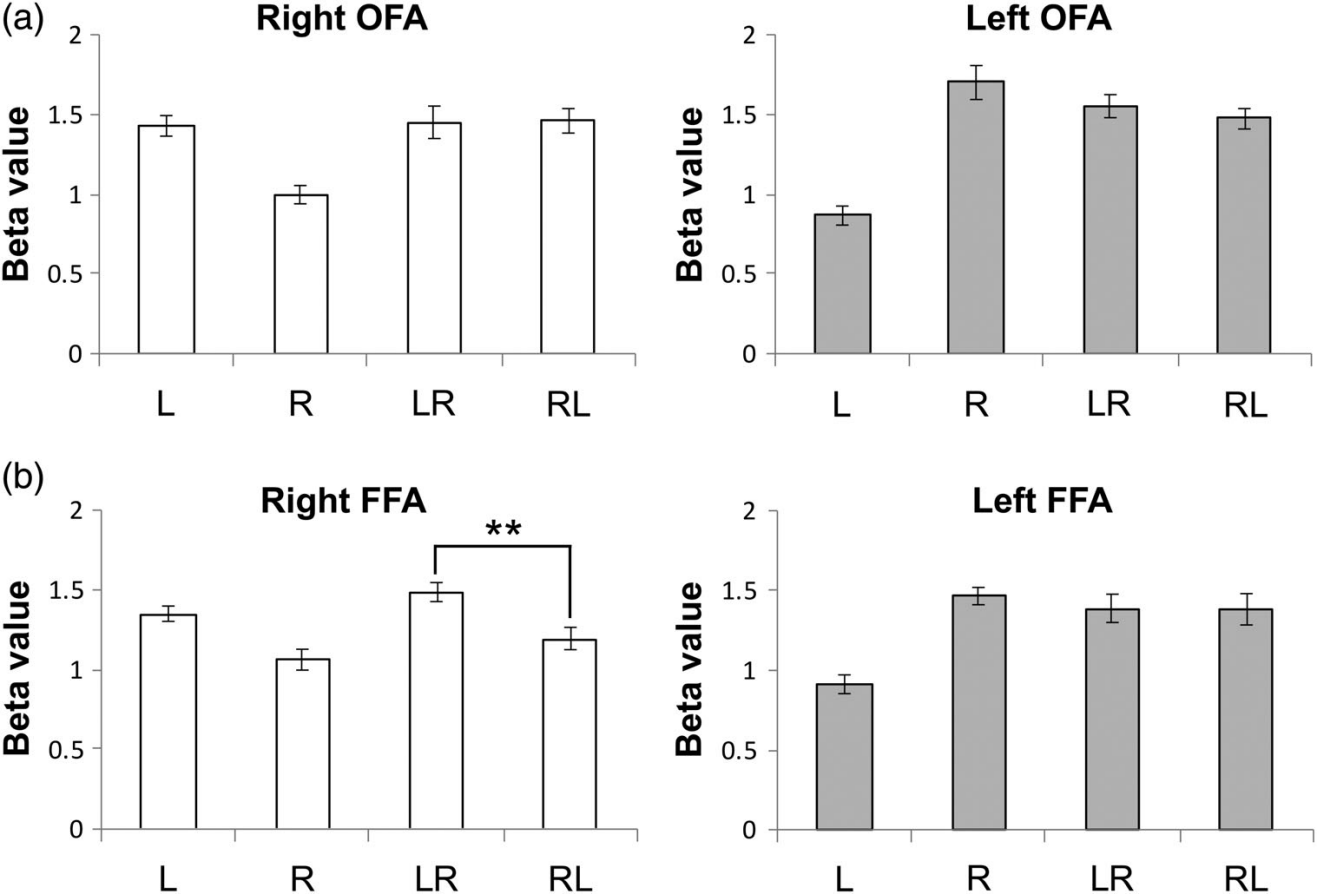
Estimated beta values from the first experiment in the OFA and FFA of both hemispheres. The only significant difference was between LR and RL in the right FFA. ** indicates *p* < .01 after correction for multiple comparisons. Error bars indicate the standard error of the mean. FFA, fusiform face area; OFA, occipital face area

The first experiment involved the use of four stimulus conditions: L (single face stimulus in the left visual field); R (single face stimulus in the right visual field); LR (paired stimuli, first face stimulus in the left visual field and second face stimulus in the right visual field); and RL (paired stimuli, first face stimulus in the right visual field and second face stimulus in the left visual field). Each condition had an ISI of 33.2 ms. Further details are supplied in the Section 2. The fMRI signals for the four conditions were extracted from the face areas of the right‐ and left FFAs and the right‐ and left OFAs and compared for the task conditions of L, R, LR, and RL. Areas other than the right FFA did not exhibit any significant differences between the task conditions at the *p* = .05 level. The responses to task conditions were significantly different (*F* = 9.03, *p* < .0001) in the right FFA, and post hoc analysis revealed a significant difference between the LR and RL responses (*p* = .006, Tukey corrected) (Figure 5). Based on the BOLD responses, the repetition suppression ratios for the LR and RL conditions were calculated according to Equation (1). The suppression ratio difference between LR and RL was 12.9% (*p* < .001, *t* test; 95% confidence interval: 9.1–16.7).

### 3.3 Measurement of repetition suppression of paired stimuli presented unilaterally in the same visual field

**Figure 6:**
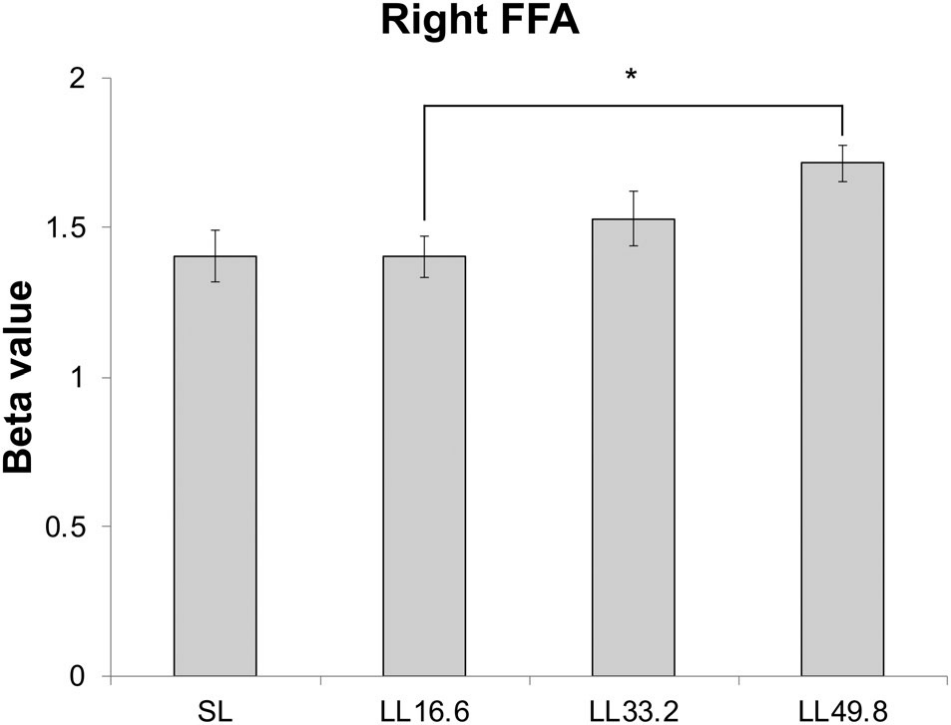
Estimated beta values from the second experiment in the FFA of the right hemisphere. There were significant differences between LL16.6 and LL49.8. * indicates *p* < .05 after correction for multiple comparisons. Error bars indicate the standard error of the mean. FFA, fusiform face area

In the second experiment, we examined ISI‐dependent responses at the right FFA by varying the ISI. The second experiment comprised four conditions: L (single face stimulus in the left visual field); LL16.6 (paired face stimuli with an ISI of 16.6 ms in the left visual field); LL33.2 (paired face stimuli with an ISI of 33.2 ms in the left visual field); and LL49.8 (paired face stimuli with an ISI of 49.8 ms in the left visual field). In the right FFA, there were significant differences between L, LL16.6, LL33.2, and LL49.8 (*F* = 3.56, *p* = .022), and post hoc analysis revealed significant differences between LL16.6 and LL49.8 (*p* = .034, Tukey corrected). The magnitude of the BOLD response increased linearly with the interval, as measured at 16.6, 33.2, and 49.8 ms (Figure 6). From the BOLD responses, the repetition suppression ratio for each condition was calculated according to Equation (3). The suppression ratio of LL16.6 was 12.3%, which was greater than that of LL49.8 (*p* < .01, *t* test; 95% confidence interval: 4.8–19.8). This 12.3% corresponded to the difference between LL16.6 and LL49.8, a 33.2‐ms ISI.

### 3.4 Derivation of a relationship between the amount of suppression and the ISI of the paired stimuli

The relationship between the ISI and response suppression was derived as follows: (a) the only difference between LL16.6 and LL49.8 was the interval length, 33.2 ms; (b) the difference of 12.3% between LL16.6 and LL49.8 was attributed to the interval difference of 33.2 ms, because the interval difference between LL16.6 and LL49.8 was 33.2 ms and the stimuli applied in LL16.6 and LL49.8 were identical; (c) the ISI between stimulus L and stimulus R in the LR and RL stimulus pairs was 33.2 ms—that is, the ISIs for LR and RL were identical; (d) the repetition suppression ratios in the right FFA for the LR and RL conditions were different; (e) the stimulus pairs LR and RL were identical, except for the order in which stimuli L and R were applied in each pair (LR and RL would be exactly the same at an ISI of zero); (f) the difference in the repetition suppression ratios between LR and RL was attributed to the transfer time between hemispheres—that is, the actual interval difference resulting from the transfer time of the signal from the left FFA to the right FFA (Figure 2); (g) the suppression ratio difference between LR and RL was 12.9%; (h) the 12.9% difference corresponded to 34.8 ms ([12.9/12.3] × 33.2), because 12.3% corresponds to 33.2 ms.

### 3.5 Estimation of transfer times between hemispheres from the suppression

The 12.9% response difference between LR and RL was twice that of the IHTT—that is, the temporal difference of LR – RL = 2 × IHTT (Figure 2); thus, the IHTT ([LR – RL]/2, i.e., 34.8 ms/2) from the left FFA to the right FFA was finally derived as 17.4 ms (95% confidence interval: 8.5–26.3, ratio statistics).

Although the main result, that is, the estimation of transfer time from the left‐hand FFA to the right FFA, was achieved, further analysis was conducted to support this result.

### 3.6 The possibility of suppression at V1

**Figure 7:**
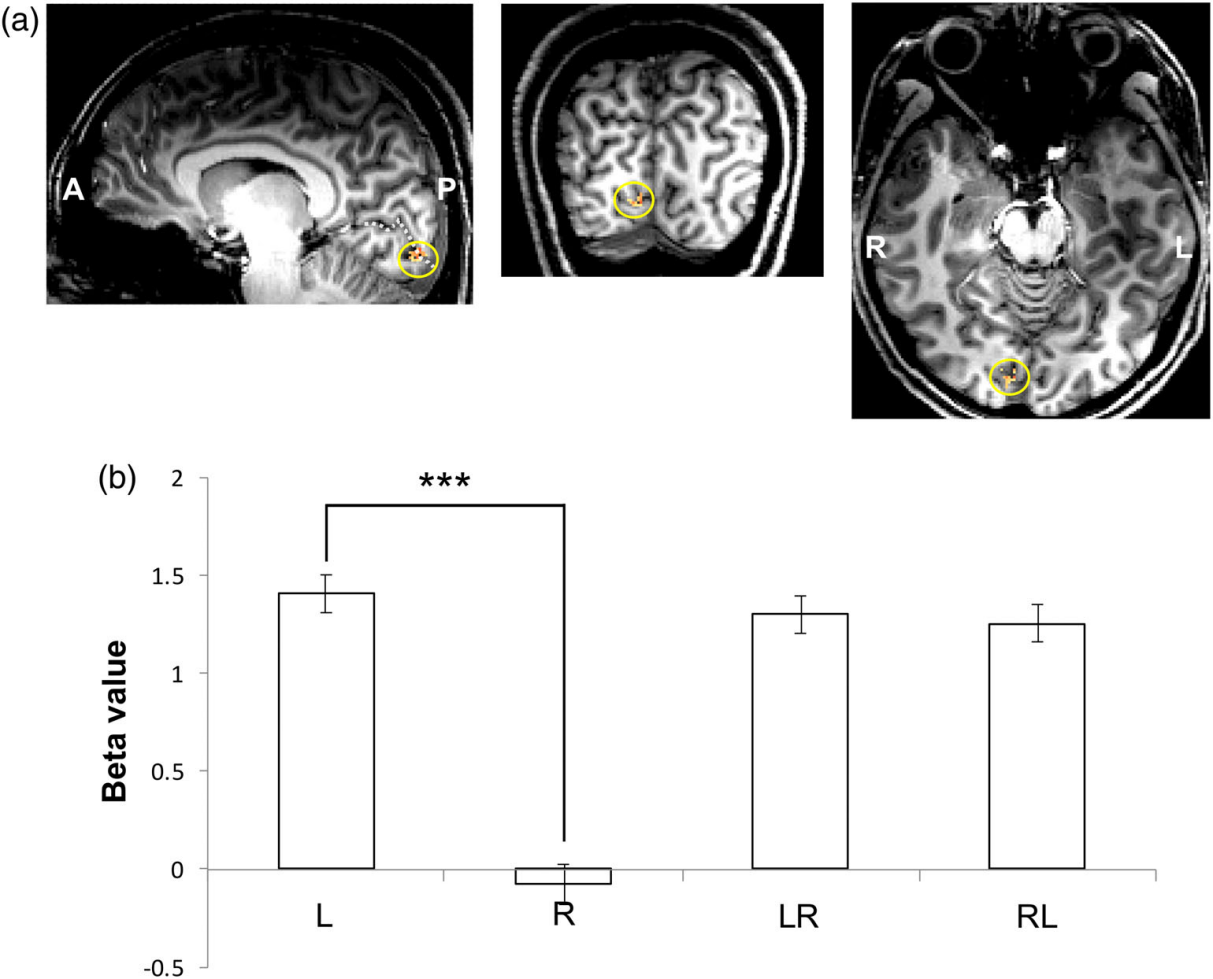
The right V1 identified by the localization experiment in the first experiment (a). Estimated beta value from the right V1 (b). The V1s from 22 subjects (*p* < .05, FDR corrected). The dotted white line in (a) indicates a calcarine sulcus in V1. *** indicates *p* < .001 after correction for multiple comparisons. Error bars indicate the standard error of the mean

In the previous study, repetition suppression was observed in the V1. We investigated whether the suppression seen in the right FFA for the paired stimuli LR and RL might be due to suppression at V1. The BOLD responses in the right V1 for L, LR, and RL were not significantly different (L, LR, and RL, *p* > .05, Tukey corrected) and they were greater than those for R (*p* < .001, Tukey corrected), suggesting an absence of repetition suppression, because the response to the ipsilateral stimulus was negligible in V1 (Figure 7).

### 3.7 The possibility of visual location‐dependent suppression

**Figure 8:**
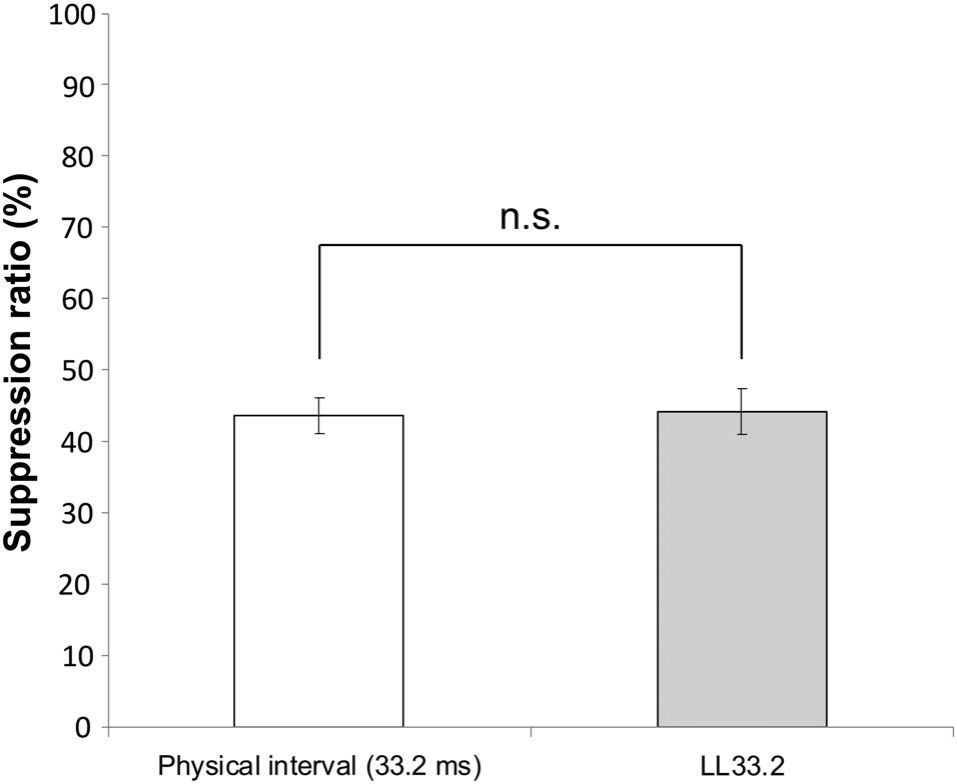
Comparison of the suppression ratio for the bilaterally presented stimuli with a transfer time of zero; that is, including only the “physical interval LR” or “physical interval RL” with an ISI of 33.2 ms (physical interval 33.2 ms), and the stimuli presented at the same locations (ISI 33.2 ms). There was no significant difference between them (*p* = .88, two‐sample *t* test). The notation n.s. indicates that the difference is not significant

We investigated whether the magnitude of repetition suppression for bilaterally presented stimuli differed from that for ipsilaterally presented stimuli at the same locations. The response suppression, when the intrinsic transfer time between the right‐ and left FFAs was zero, could be derived from the BOLD responses of LR and RL (Equations 4 and 5). The derived response suppression, which corresponded to the suppression of bilaterally presented stimuli, was 43.7% (*p* < .001, 95% confidence interval: 38.5–48.8). This value was not significantly different (*p* = .88, two‐sample *t* test) from the suppression that occurred with the ipsilaterally presented stimuli of ISI 33.2, 44.3% (*p* < .001, 95% confidence interval: 37.3–51.2) (Figure 8).

### 3.8 The estimation of experiment‐dependent BOLD response

**Figure 9:**
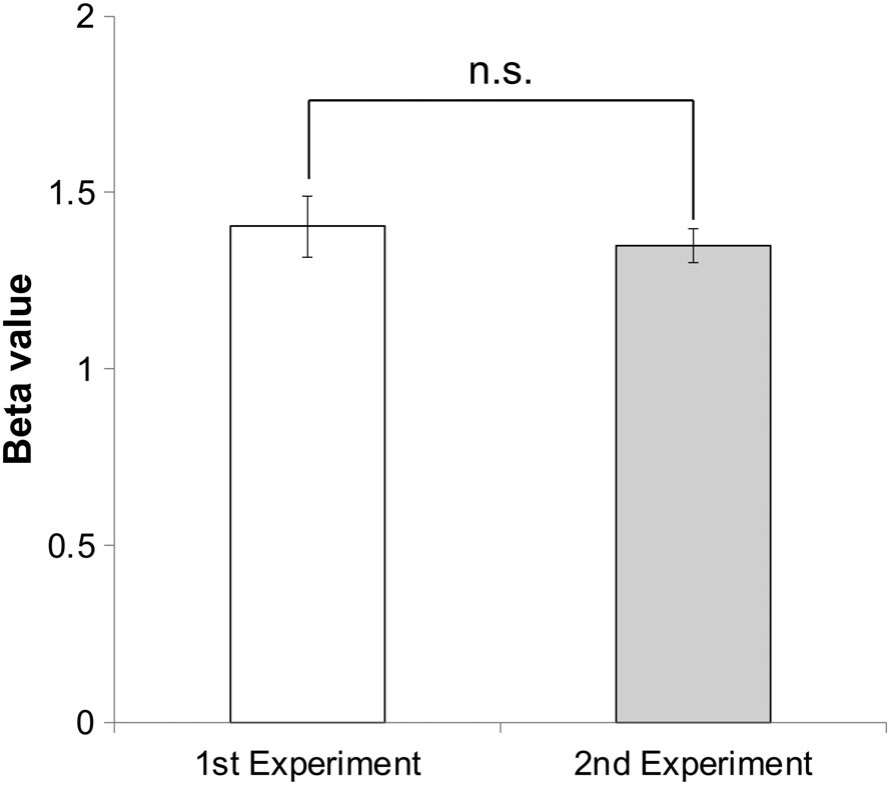
Comparison of beta values for stimulus condition L at the right V1 between the first and second experiments (*p* = .59). The notation n.s. indicates that the difference is not significant

As the estimation of the transfer time was based on two experiments, we assessed the difference in the BOLD response at the right V1 between the two experiments with respect to stimulus condition L, which was common to the two experiments. This comparison revealed that the BOLD response did not differ significantly between the two experiments (*p* = .59, two‐sample *t* test; Figure 9).

## 4 DISCUSSION

The present study aimed to assess the feasibility of using fMRI to measure the transfer time between homotopic brain areas related to face processing. We could measure the transfer time by the repetition suppression of BOLD responses using a novel ISI stimulation paradigm. Our measured IHTT is similar to that reported in EEG studies (Barnett & Corballis, 2005; Brown, Larson, & Jeeves, 1994), indicating that the transfer time measured by fMRI is reasonable.

We found that, in the same visual field, responses to the paired conditions LL16.6, LL33.2, and LL49.8 increased linearly in the right FFA. The conditions differed only in the duration of the ISI, because the same stimulus was presented twice with each ISI for each condition. Therefore, the BOLD response difference between the conditions reflects the interval difference. Previous fMRI studies have shown that suppression of the BOLD response varies depending on the ISI when a stimulus is presented twice (Ogawa et al., 2000; Sung et al., 2010), and response suppression of this type reflects the neuronal responses to stimuli arriving sequentially at a functional site, which can be interpreted as repetition suppression, repeated suppression, fMRI adaptation, and more (Grill‐Spector, Henson, & Martin, 2006; Larsson & Smith, 2012; Ogawa et al., 2000), although there may be differences in the details of these phenomena. Similar response suppression has been seen in EEG and MEG experiments (Engell & McCarthy, 2014; Garrido et al., 2009).

The difference in BOLD response suppression between LL16.6 and LL49.8 is thought to be due to the difference in the ISI (33.2 ms), as this is the only difference between these conditions. Similarly, in the case of LR and RL, the physical (explicit) ISI is identical at 33.2 ms, and the only difference is the order of presentation of the two identical face images in the stimulus pairs. In addition, the FFA receives the signal inputs irrespective of the visual field, as demonstrated in earlier studies (Sung et al., 2010). The observation that response suppression by bilaterally presented stimuli, when the intrinsic transfer time between the right‐ and left FFAs was zero, did not differ significantly from the response suppression by ipsilaterally presented stimuli (Figure 8) shows that the same suppression occurs at the FFA for both bilaterally and ipsilaterally presented stimuli. Therefore, the only difference at the right FFA between the LR and RL conditions is the difference in putative (implicit) intervals arising from the transfer time of the signals between the hemispheres. These findings show that the responses of LR and RL are modulated solely by the actual ISI, including the transfer time, and exclude the possibility that other effects, such as the laterality of visual presentation in either bilateral or ipsilateral visual fields, are involved. Our previous studies have shown that there was no significant difference (*p* = .79, *t* test) in repetition suppression at FFA with an ISI of 132 ms by stimuli presented at the same locations and those by stimuli at different locations, both in the same visual hemifield (Figure S1). These data also support the theory that repetition suppression by bilaterally presented stimuli is the same as the suppression by stimuli presented at the same location, which also supports our IHTT calculation.

Several previous electrophysiological studies showed that interhemispheric responses have latencies of tens of milliseconds between contralateral hemispheres of rats or monkeys (Seggie & Berry, 1972; Swadlow, Rosene, & Waxman, 1978; Weber et al., 2008). Further, IHTT varies between species, for example, 2.6–18.0 ms in monkey and 2.4–39.8 ms in rabbit (Swadlow et al., 1978); IHTT measured in our study was within the range observed in previous studies. The variation of IHTT is known to be caused by different proportions of axon myelination in the corpus callosum (CC), interhemispheric conduction distance, and axon diameters of CC (Phillips et al., 2015). Measurement of these factors in relation with our measured IHTT may be performed through diffusion MRI, which will be the focus of our future study.

From the observation that repetition suppression depends solely on the ISI, the same difference is expected in the left FFA if the transfer time between hemispheres is symmetrical from left to right and from right to left. Therefore, the finding that the BOLD response between LR and RL differed only in the right FFA suggests that the transfer time is not symmetrical. Although some physiological or physical factors, such as the BOLD signal responses and measurement noise, may contribute to the difference, the difference between LR and RL does not seem to be due to SNR differences related to the intrinsic BOLD signal in the right‐ and left FFAs, because a comparison of the values of the temporal SNR at the right‐ and left FFAs reveals no statistically significant difference (*p* = .76, paired *t* test). Therefore, the relationship between LR and RL at the right‐ and left FFAs can be interpreted as indicating that the signal transfer time between the hemispheres is asymmetrical at the FFA level. Lateralization of the IHTT in visual areas has been reported in previous EEG studies (Barnett & Corballis, 2005; Brown et al., 1994), which showed that the transfer time from right to left hemispheres was shorter than that from left to right hemispheres. In addition, a recent fMRI study has revealed differences in the cortical layer involving the propagation of slow spontaneous activities. This suggests a similar asymmetry, as the spontaneous activity may occur in fast signal processing if the fast stimuli‐driven signal processing is based on the signal processing mechanism in the resting state (Mitra et al., 2018). The existence of this kind of asymmetry is further supported by an asymmetry seen in white matter structure, in which the volume of the right inferior longitudinal fasciculus was observed to be larger than that of the left ILF (Latini et al., 2017), supporting our inference of asymmetry in the IHTT.

As the difference between LR and RL was not significant in the left FFA and is considered to be due to shorter transfer times from the right to the left FFA, a similar inference may be applied to the OFA. The transfer time at the OFA level may be shorter than that at the FFA level. Further studies are required to measure the transfer time at the OFA level. This will necessitate overcoming several problems, such as the need for a shorter presentation time for stimulation and for more precise fMRI measurements. The volume of signal transferred may be another reason for the absence of BOLD response differences at the OFA level. The OFA is located at a lower level of the hierarchical structure of the face‐processing flow, and the amount of signal transferred at the OFA level is almost half of that transferred at the FFA level (Choi et al., 2013), a finding that should be confirmed in future studies.

In considering the hierarchical structure of the brain in face signal processing, one may consider that repetition suppression occurs in V1 transfers to the FFA. A previous study using a simple block design paradigm comprising left or right visual hemifield stimulation blocks has shown that the response in V1 to the ipsilateral stimulus was almost zero (Choi et al., 2013). This finding was replicated in the current study, as observed in the responses in V1 to L, R, RL, and LR, where the responses were from the contralateral stimulus alone; that is, L ≅ RL ≅ LR and R ≅ 0 (Figure 7). This finding can exclude the possibility of signal modulation at the FFA by suppression at V1. In addition, the lack of a significant difference between LR and RL in the OFA, meaning that the signal difference in the FFA between LR and RL was caused at least after the OFA level, also supports the idea that signal modulation in the FFA is not due to modulation in cross‐hemispheric connections between the V1s. In addition, signal modulation at FFA by signals from high level areas was dismissed by our previous 3T experiments with the same stimuli of Face and Scene as used in this study confirmed that only OFA and FFA under the passive viewing task activate without showing any other face areas (Figure S2). In association with repetition suppression of FFA, one may also consider the possibility of response modulation at early visual areas by a flickering effect (or frequency modulation) in the case of stimuli with very short ISIs, such as in the second experiment. However, several studies using face stimuli have demonstrated that the frequency modulation, especially at high frequencies, disappears in high‐tier visual areas, such as the FFA (Gauthier, Eger, Hesselmann, Giraud, & Kleinschmidt, 2012; Gentile & Rossion, 2014).

In this study, data from different subject groups were employed to calculate the IHTT. However, the comparison of the BOLD responses between the L conditions of the first and second experiments shows that there were no significant differences between them and eliminates the possibility that our results can be attributed merely to differences between subject groups (Figure 9).

We used the intertrial interval (ITI) of 1.5 s in this study for the complete recovery of the response suppression of neuronal origins because the response suppression is known to be recovered at around 1 s (Ogawa et al., 2000). However, a previous study reported that some nonlinearity even at ITIs greater than 1 s originates from vasculature (Zhang, Liu, He, & Chen, 2008). Moreover, nonlinearity was known to be largely alleviated after removing large vessels (Zhang, Liu, et al., 2008; Zhang, Zhu, & Chen, 2008). These previous studies may indicate that our observed amplitudes of BOLD signals may be modulated by ITI and affect our results. However, the ISIs of our study were very short (49.8 ms for the longest one) and were negligible compared with the ITI. In addition, the same ITI was used for all condition blocks; therefore, the effects could be canceled out between conditions in the IHTT calculation. Further, a previous study showed that large vessels do not pass through FFA (Chung, Sung, & Ogawa, 2015). Thus, the nonlinearity of vasculature origin observed in the previous studies would not affect our results.

The IHTT is an important factor to understand visual information processing; however, its characteristics have not yet been elucidated using conventional fMRI. Although a deconvolution analysis of the BOLD signal has been employed to evaluate brain function with a temporal resolution of several seconds (Lewis et al., 2018), this approach has limitations, including a low SNR and dependency on the deconvolution of similar time courses. By contrast, our study demonstrated the feasibility of using fMRI to measure the temporal characteristics of neuronal activity of visual processing at a temporal resolution of tens of milliseconds. Recent trends toward ultra‐high spatial resolution studies may allow us to measure the transfer times between cortical layers, as observed in the resting‐state study (Mitra et al., 2018), and to elucidate brain dynamics at the cortical layer level. This is a major aim of our future work.

We measured the IHTT at the FFA level, assuming a very short transfer time and a relationship between repetition suppression through significant differences in the BOLD response, although this is not a direct measurement of neuronal progress. However, our proposed method would be useful in elucidating brain dynamics, given that there is no method to directly measure the IHTT between specific brain areas. Although high temporal neuroimaging modalities, such as EEG or MEG, can record the characteristics of signal transfer with millisecond resolution (Engell & McCarthy, 2014), even these modalities cannot provide accurate spatial information because of their low spatial resolution and source reconstruction problems (Michel et al., 2004). Many research groups have used either fMRI–EEG or fMRI–MEG integration methods to determine both accurate anatomical locations and fast temporal characteristics of brain functions (Jorge, van der Zwaag, & Figueiredo, 2014). They have used these methods to measure very accurate signal transfer times, in the order of tens of milliseconds, between specific brain areas (Baldauf & Desimone, 2014). The combination of these methods and our methods may provide a valuable tool for future studies measuring transfer times at a cortical laminar level.

Our findings show that it is feasible to use 7T fMRI to measure the temporal characteristics of neuronal activity in the brain at a millisecond resolution. We expect these findings to pave the way to further understand dynamic brain systems.

## 5 CONCLUSIONS

We devised a novel stimulus paradigm and could measure signal transfer time between hemispheres. Our method and findings demonstrate the feasibility of using fMRI to measure ultra‐fast signals (tens of milliseconds) and could facilitate the elucidation of further aspects of dynamic brain function.

## Supporting information


**Appendix** S1: Supplementary Information

## Data Availability

Data sharing is not applicable to this article as no new data were created or analyzed in this study.
