# Supplementary material for: Measurement of ultra‐fast signal progression related to face processing by 7T fMRI
Source: Hum Brain Mapp. 2020 Jan 10;41(7):1754–64. doi: 10.1002/hbm.24907 (PMC7268038; doi:10.1002/hbm.24907)
Supplement: Supplementary file 1 — Appendix S1: Supplementary Information [file HBM-41-1754-s001.docx]

Supplementary
